# Holopatient technology in nursing education: a cross-sectional analysis of student and faculty perceptions

**DOI:** 10.1186/s12912-025-03856-6

**Published:** 2025-09-26

**Authors:** Amal Ali Alharbi, Wesam Taher Almagharbeh, Hazem AbdulKareem Alfanash, Khaldoon Aied Alnawafleh, Amal Ali Alasmari, Sameer A. Alkubati, Malik A. Altayar, Nesreen AbdelMonaem AbouZeid, Khulud Ahmad Rezq, Elham H. Othman

**Affiliations:** 1 Department of Nursing Administration and Education, Faculty of Nursing, University of Tabuk, Tabuk, Saudi Arabia; 2 Medical and Surgical Nursing Department, Faculty of Nursing, University of Tabuk, Tabuk, Saudi Arabia; 3 Department of Medical Surgical Nursing, College of Nursing, University of Ha’il, Ha’il City, Saudi Arabia; 4 Department of Medical Laboratory Technology, Faculty of Applied Medical Sciences, University of Tabuk, Tabuk, Saudi Arabia; 5 Department of Medical Surgical Nursing, College of Nursing, Princess Nourah bint Abdulrahman University, P.O. Box 84428, Riyadh, 11671 Saudi Arabia; 6 Community & Psychiatric health Nursing department, Faculty of Nursing, University of Tabuk, Tabuk, Saudi Arabia; 7 Faculty of Nursing, Applied Science Private University, Amman, Jordan; 8 Faculty of Nursing, Jerash University, Jerash, Jordan

**Keywords:** Holopatient simulation, Nursing education, Mixed reality, Digital literacy, Technology adoption

## Abstract

**Background:**

Holopatient technology, a mixed reality simulation tool, is increasingly used in nursing education to enhance clinical reasoning and student engagement. However, differences in perception between students and faculty remain underexplored.

**Methods:**

A cross-sectional survey was conducted among 126 participants (84 nursing students and 42 faculty members) at a Saudi Arabian nursing college. All participants had prior exposure to Holopatient scenarios, including adult medical-surgical and maternal health cases. A researcher-developed, 20-item Likert-scale questionnaire assessed four domains: perceived effectiveness, overall satisfaction, ease of use, and implementation challenges. Descriptive statistics, chi-square tests, and multiple regression analyses were performed.

**Results:**

Students rated perceived effectiveness significantly higher (mean [*M*] = 4.2, standard deviation [*SD*] = 0.6) than faculty (*M* = 3.8, *SD* = 0.8; *p* < 0.01). Satisfaction was also higher among students (*M* = 4.1, *SD* = 0.7) than faculty (*M* = 3.7, *SD* = 0.9). Ease of use was similar (*M* = 4.0 vs. 3.9), while faculty reported more implementation challenges (*M* = 3.5, *SD* = 0.9) than students (*M* = 3.0, *SD* = 0.6). A significant association was found between faculty teaching experience and perceived challenges (χ^2^ (4) = 15.78, *p* < 0.05). Regression analysis showed that prior simulation exposure (β = 0.35, *p* < 0.001), digital literacy (β = 0.28, *p* = 0.01), and fewer teaching years (β = −0.15, *p* = 0.05) predicted more positive perceptions. The model explained 35% of the variance (*R*^*2*^ = 0.35).

**Conclusions:**

Students expressed more favourable perceptions of Holopatient technology than faculty. Digital literacy and prior simulation exposure were strong predictors of acceptance, while longer teaching experience was linked to lower enthusiasm. Faculty development and targeted support are essential for successful adoption.

**Clinical trial number:**

Not applicable.

**Supplementary Information:**

The online version contains supplementary material available at 10.1186/s12912-025-03856-6.

## Background

Advancements in healthcare education increasingly incorporate simulation technologies to enhance clinical competence, critical thinking, and student engagement [1, 2]. Among these innovations, Holopatient technology—an augmented and mixed reality-based tool—offers immersive, holographic patient simulations that allow nursing students to interact with lifelike clinical scenarios in a controlled, safe environment [3, 4]. Recognized by the World Health Organization as a key strategy for improving nursing education, particularly in low-resource settings, Holopatient technology has been increasingly adopted in high-income countries such as the United Kingdom and Australia [5–7].

Despite its promise, disparities in adoption and implementation remain. High setup costs, ranging from $50,000 to $200,000, along with the need for technical training and infrastructure, hindered widespread use, especially in low- and middle-income countries [8, 9]. Even in institutions where Holopatient simulations are integrated, differing perceptions among stakeholders persisted. Kang and Lee (2023) found that nursing students perceive mixed reality-based Holopatient simulations as highly effective in enhancing their understanding of clinical scenarios and improving learning outcomes [4]. Similarly, Son, Kang, and De Gagne (2023) observed that students who used Holopatient during the COVID-19 pandemic reported increased self-confidence, satisfaction, and motivation [10].

Previous studies emphasized the benefits of augmented reality in nursing education, including improved information retention, empathy, and teamwork [11–13]. However, few directly compared how students and faculty perceived Holopatient technology in terms of effectiveness, usability, and implementation barriers. Given that faculty play a critical role in curricular integration, their perspectives is vital to the technology’s sustained use.

This study aimed to address that gap by conducting a cross-sectional comparison of nursing students’ and faculty members’ perceptions of Holopatient technology. It also sought to identify predictors of acceptance, offering practical insights for enhancing simulation-based learning in nursing education.

## Methods

### Design and setting

This study employed a quantitative, cross-sectional design to investigate the perceptions of nursing students and faculty regarding the use of Holopatient technology in nursing education. The study was conducted within a large nursing education program where Holopatient simulations had recently been introduced as part of the curriculum. Holopatient technology was integrated into clinical simulation modules for adult medical-surgical and maternal-child health courses, using mixed-reality devices such as HoloLens headsets (Microsoft Corporation, Redmond, WA). “Digital tools” in this context referred to educational technologies, including virtual simulations, online learning platforms, and basic interactive systems that support digital engagement in teaching and learning. Holopatient simulations were piloted in Fall 2023 and fully integrated into lab-based teaching activities during the Spring 2024 semester.

Ethical approval for the study was obtained from the Institutional Review Board (IRB) of the participating institution. All participants were informed about the purpose of the study and provided written informed consent prior to data collection. Participation was voluntary, and respondents were assured of confidentiality and anonymity. Data were de-identified and analyzed in aggregate to protect individual privacy. Only participants with prior exposure to Holopatient technology and a basic understanding of digital tools were eligible to take part in the study.

## Participants and sampling

A total of 126 participants were included in the study, comprising 84 nursing students and 42 nursing faculty members. All were recruited from a single institution where Holopatient simulation had recently been introduced into the curriculum. Informed consent was obtained from all participants after they were provided with a detailed information sheet explaining the study’s purpose, procedures, potential risks and benefits, and their right to withdraw at any time without penalty.

A purposive sampling method was employed to ensure the representation of individuals with varying levels of experience and familiarity with digital and simulation tools. Eligibility criteria included: (1) current enrollment in the nursing program for students, or active teaching status for faculty members; (2) prior exposure to Holopatient technology; and (3) basic competence with digital learning tools. Digital competence was self-reported using a short checklist confirming prior use of educational technologies, including virtual simulations and online platforms. Participants who were not directly involved in nursing education or had no prior experience with Holopatient simulations were excluded from the study.

To determine the adequacy of the sample size, a power analysis was conducted, ensuring sufficient statistical power (0.80) to detect significant differences between the student and faculty groups. This analysis confirmed that a minimum of 120 participants was appropriate to support the use of independent samples t-tests and regression analyses.

## Data collection and variables

### Questionnaire design and validation

A researcher-developed, structured questionnaire was used for both nursing students and faculty members. The same core instrument was administered to both groups, with minor wording adjustments to reflect role-specific perspectives. For instance, an item for students read, “Holopatient technology enhanced my clinical reasoning skills,” while the corresponding item for faculty read, “Holopatient technology enhanced student clinical reasoning skills.” Similarly, questions related to implementation challenges were phrased to reflect faculty responsibilities such as curricular integration, while students were asked about their individual learning experience. The questionnaire included 20 items across four domains: perceived effectiveness, overall satisfaction, ease of use, and implementation challenges. A 5-point Likert scale was used to rate all items (1 = strongly disagree to 5 = strongly agree). The full questionnaire is available in the Supplementary Materials.

Survey items were developed based on prior literature on simulation-based learning and augmented reality in nursing education. The structure and content were informed by previously validated instruments in related studies and refined through expert review by faculty with experience in digital education and simulation pedagogy.

A preliminary test of the questionnaire took place through pretesting with nursing students and faculty members to maintain clarity and achieve both comprehensive content and valid measurement. The pilot phase feedback led to minor changes in both wording and layout of questionnaire items. A group of expert reviewers who specialize in nursing education and digital learning technologies assessed the instrument for content validity while checking its alignment with the constructs studied in relevant literature. Due to the exploratory nature of the study and the moderate sample size, exploratory or confirmatory factor analysis was not performed. Instead, internal consistency reliability was assessed using Cronbach’s alpha coefficients, which demonstrated high levels of reliability across all domains: perceived effectiveness (α = 0.85), overall satisfaction (α = 0.83), ease of use (α = 0.82), and implementation challenges (α = 0.84). Future studies will incorporate factor analytic validation.

### Variables

The study collected data on both demographic and perception-related variables. Demographic information included participants’ age, gender, profsional role (student or faculty), years of teaching experience in the case of faculty, and prior exposure to digital tools. Faculty were also asked to self-report their area of specialization (e.g., medical-surgical nursing, nursing education, community/mental health) as part of the demographic questionnaire. Digital literacy was measured using a self-reported item where participants rated their proficiency with digital education technologies on a 5-point Likert scale (1 = very low to 5 = very high).

Perception-related variables were grouped into four domains. Perceived effectiveness was measured by examining how participants evaluated the impact of Holopatient technology on learning outcomes, clinical reasoning, and knowledge retention. Overall satisfaction captured the degree of enjoyment, engagement, and general approval of the learning experience provided by the technology. Ease of use referred to participants’ experiences with the interface, including its intuitiveness, accessibility, and the ease with which users could navigate the simulation environment. Implementation challenges reflected difficulties in integrating the technology into educational practices, including technical barriers, curriculum alignment issues, and the need for faculty training.

## Statistical analysis

SPSS software version 26 (IBM Corp., Armonk, NY, USA) was used to perform statistical analysis. Descriptive statistics were first calculated to summarize the demographic characteristics of the participants and their responses across the four measured domains: perceived effectiveness, overall satisfaction, ease of use, and implementation challenges. Means and standard deviations were reported for each variable, allowing for comparison between nursing students and faculty members.

To test for statistically significant differences between groups, an independent samples t-test was used to compare mean scores for perceived effectiveness, satisfaction, and ease of use between students and faculty. A chi-square test of independence was conducted to assess the association between years of teaching experience and perceived implementation challenges among faculty.

Additionally, multiple regression analysis was employed to identify predictors of positive perceptions toward Holopatient technology. The dependent variable—overall positive perception—was computed as the average of the four domain scores (perceived effectiveness, satisfaction, ease of use, and implementation challenges). Items in the implementation challenge domain were reverse-coded prior to averaging to ensure directionality aligned with positive perception. Predictor variables included prior exposure to digital tools, digital literacy, years of teaching experience, and area of specialization. Regression assumptions, including linearity, normality, and homoscedasticity of residuals, were checked prior to analysis.

Statistical significance was set at *p* < 0.05 for all tests. Effect sizes (e.g., Cohen’s d for t-tests and standardized beta coefficients for regression analysis) were reported to assess the practical significance of the findings.

## Results

### Participants characteristics

The study included 126 participants, consisting of 84 nursing students and 42 faculty members. Among the students, 50 (59.5%) were aged between 20 and 25 years. In the faculty group, 29 (69.0%) were aged between 35 and 50 years. Most participants were female, comprising 95 (75.4%) of the total sample. Regarding teaching experience, 32 (76.1%) of faculty members reported having less than 10 years of teaching experience. Students reported prior exposure to digital tools more frequently than faculty, suggesting a higher baseline of digital familiarity among the students’ group (Table 1).

**Table 1:** Demographic characteristics of the participants (*N* = 126)

Characteristic	Students (N = 84)	Faculty (N = 42)
**Age Group**		
20–25 years, N(%)	50 (59.5%)	4 (9.5%)
25–30 years, N(%)	31 (36.9%)	29 (69.1%)
35–50 years, N(%)	3 (3.6%)	9 (21.4%)
**Sex**		
Female, N(%)	67 (79.8%)	29 (69.0%)
Male,N(%)	17 (20.2%)	13 (31.0%)
**Years of Teaching Experience**		
< 5 years, N(%)	N/A	17 (40.5%)
5–10 years, N(%)	N/A	15 (35.7%)
> 10 years, N(%)	N/A	10 (23.8%)
**Prior Exposure to digital Tools**	71 (84.5%)	25 (59.5%)

### Perceptions of holopatient technology

Across all participants, the general perceived effectiveness of Holopatient technology was rated positively, with a combined mean (SD) of 4.0 (0.7). When analyzed by group, nursing students reported higher scores across most domains compared to faculty. Students rated perceived effectiveness at 4.2 (0.6), overall satisfaction at 4.1 (0.7), ease of use at 4.0 (0.5), and implementation challenges at 3.0 (0.6). Faculty rated perceived effectiveness at 3.8 (0.8), overall satisfaction at 3.7 (0.9), ease of use at 3.9 (0.7), and implementation challenges at 3.5 (0.9).

### Group differences in perceptions

**Fig. 1:**
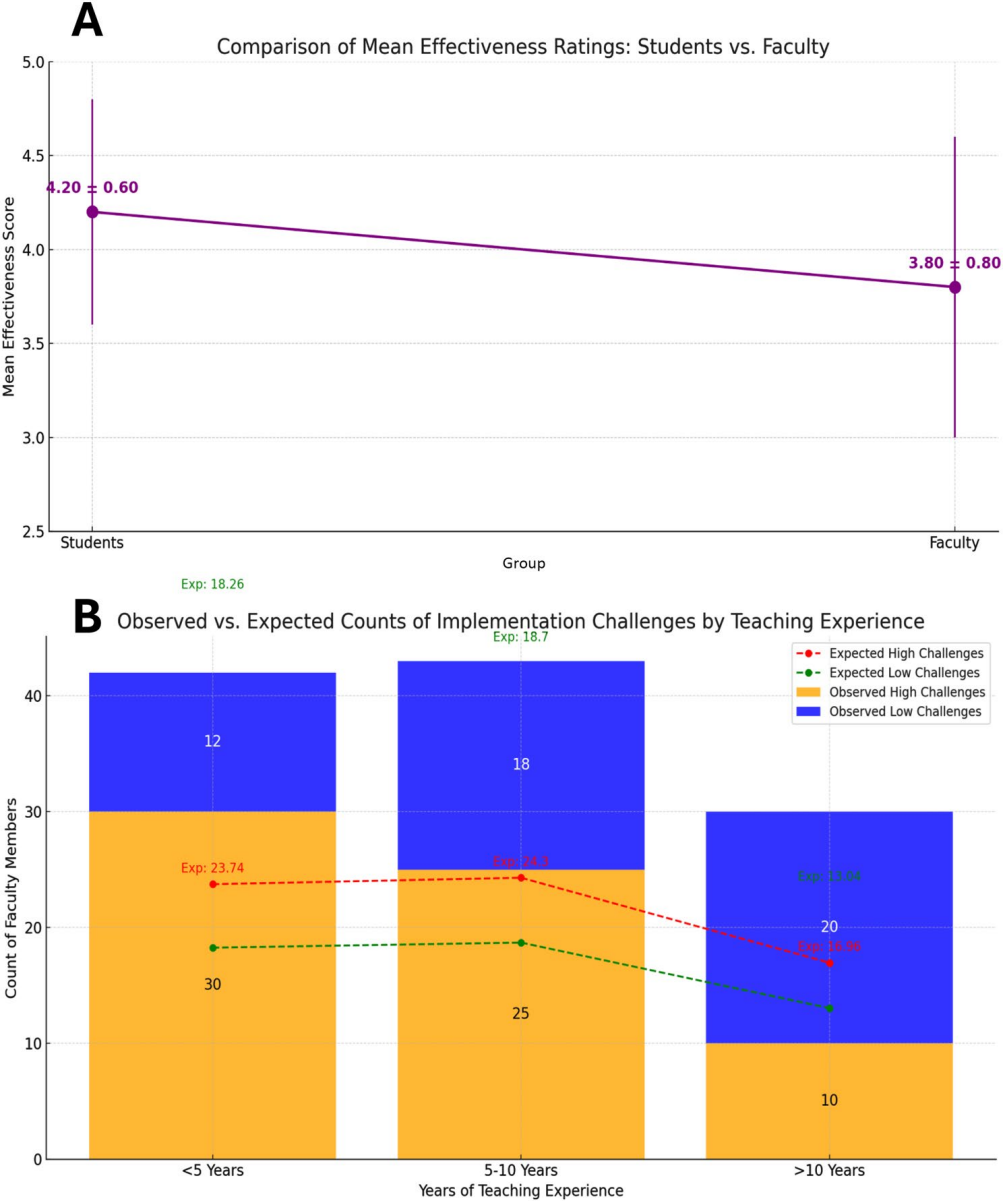
(**A**) comparison of mean effectiveness ratings for students and faculty; (**B**) perceptions of implementation challenges by teaching experience

An independent samples t-test was conducted to compare the perceived effectiveness of Holopatient technology between nursing students and faculty members. Students reported a significantly higher mean (SD) score of 4.2 (0.6) compared to 3.8 (0.8) among faculty. The difference between groups was statistically significant, t (124) = 3.45, *p* < 0.01. The calculated effect size (Cohen’s d) was 0.55, indicating a medium effect (Fig. 1A).

A chi-square test of independence was used to examine the association between years of teaching experience and perceived implementation challenges. The results showed a statistically significant association between these variables, χ^2^ (4) = 15.78, *p* < 0.05. Faculty members with less than 5 years of teaching experience reported the highest levels of perceived challenges. Residuals from the observed and expected counts were based on responses to questionnaire items related to implementation challenges. Those with less experience reported more challenges than expected, while those with more than 10 years of experience reported fewer. (Fig. 1B).

### Predictors of positive perceptions

**Fig. 2:**
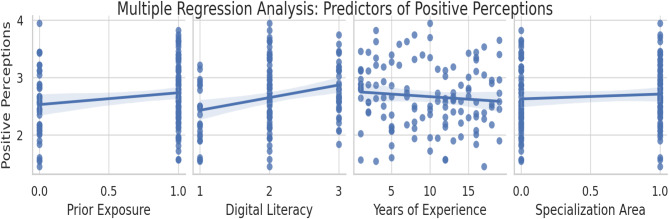
Predictors of positive perception toward holopatient technology among faculty and students. The multiple regression model included three significant predictors: prior simulation exposure (β = 0.35, *p* < 0.001), digital literacy (β = 0.28, *p* = 0.01), and teaching experience (β = −0.15, *p* = 0.05). The model accounted for 35% of the variance in overall perception (R^2^ = 0.35)

A multiple linear regression analysis was conducted to examine the predictors of overall positive perceptions toward Holopatient technology. The regression model included four predictors: prior exposure to digital tools, digital literacy, years of teaching experience, and specialization area. The dependent variable was the overall positive perception score, measured on a 5-point Likert scale. The overall model was statistically significant, F (4, 121) = 18.24, *p* < 0.001, and explained 35% of the variance in positive perceptions (R^2^ = 0.35). Three predictors were statistically significant. Prior exposure to digital tools was the strongest predictor (β = 0.35, SE = 0.10, *p* < 0.001), followed by digital literacy (β = 0.28, SE = 0.08, *p* = 0.01), and years of teaching experience (β = −0.15, SE = 0.07, *p* = 0.05). The specialization area was not a significant predictor (β = 0.05, SE = 0.09, *p* = 0.32) (Fig. 2).

## Discussion

This study aimed to explore and compare perceptions of Holopatient technology between nursing students and faculty, while also identifying key factors influencing these attitudes. Three key findings emerged. First, students consistently viewed technology more favorably than faculty, suggesting a generational or experiential divide in receptiveness to immersive digital tools. Second, faculty members, particularly those early in their teaching careers, reported greater challenges with implementation, reflecting possible gaps in training or institutional support. Third, individuals with prior exposure to simulation-based learning and higher digital literacy tended to express more positive perceptions overall, while those with longer teaching experience appeared more reserved in their evaluations. Together, these findings underscore the complex interplay between experience, familiarity, and openness to educational innovation.

Several findings of this study are consistent with the broader body of literature examining the role of Holopatient and mixed reality technologies in nursing education. Like the current results, Rummel et al. (2023) reported that students tend to rate augmented reality simulations more favorably than faculty, likely due to their familiarity with interactive and immersive digital platforms [3]. Similarly, Kang and Lee (2023) found that mixed reality learning tools increased student engagement and critical thinking, aligning with the higher satisfaction levels reported by students in this study [4].

On the other hand, faculty members in prior research have expressed concerns over integration difficulties and the steep learning curve associated with using Holopatient systems, echoing the implementation challenges and lower satisfaction observed among faculty participants here [11, 12]. Studies by Rincon et al. (2023) and Pregowska et al. (2022) also noted that technical complexity and lack of prior experience were significant barriers for instructors, particularly those newer to simulation-based teaching methods [12, 14].

Interestingly, our results showed that faculty members with less than five years of teaching experience reported greater implementation challenges compared to their more experienced colleagues. At first glance, this may seem counterintuitive since early-career faculty are often presumed to be more digitally fluent. However, literature on technology integration in nursing education and higher education highlights that digital competency alone does not ensure successful adoption. For instance, nurse educators have reported feeling overwhelmed by demands on their time and instructional planning when learning to use technologies amid broader transitional challenges early in their careers [15]. Moreover, research into higher education faculty’s attitudes toward technology suggests that new instructors often face an especially steep “cognitive load”—balancing curricular responsibilities, establishing instructional identities, and navigating institutional expectations—which can limit their capacity for adopting novel educational tools. This context may account for our finding, as less experienced faculty may struggle with multiple role stressors that outweigh enthusiasm for digital innovation. We suggest that implementing targeted professional development and mentoring, especially for early-career faculty, could reduce this cognitive burden and facilitate holistic integration of immersive simulation technologies.

These findings reveal a nuanced pattern: faculty with fewer years of teaching experience reported greater implementation challenges, likely due to role-transition burdens that dilute their capacity for educational innovation. This aligns with cognitive load theory, which suggests that early-career professionals face heightened mental burdens while adapting to new roles and responsibilities [16].

At the same time, faculty with more years of experience tended to express less enthusiasm for Holopatient technology, as reflected in lower perceived effectiveness and satisfaction scores. This finding is consistent with Kotcherlakota et al. (2017), who found that faculty experience inversely correlated with technology usage confidence and innovation adoption in nursing education [17].

Moreover, our regression findings reinforce previous research identifying digital literacy and prior exposure to digital tools as key determinants of user acceptance. Minty et al. (2022) highlighted that digital fluency strongly predicts educators’ willingness to adopt simulation-based methods, while Zarakovitis and Tsoromokos (2024) emphasized that resistance among experienced faculty remains a persistent barrier to innovation despite the benefits of immersive technologies [8, 18]. The significance of teaching experience as a negative predictor in this study resonates with the broader observation that seasoned educators may be less adaptable to rapidly evolving technologies [13, 18, 19]. Additionally, Choi and Kim’s (2024) findings support the idea that team-based, simulation-driven education improves student satisfaction, yet often requires substantial institutional investment and faculty training—factors that continue to influence adoption rates globally [13].

While the regression model identified key predictors, it explained only 35% of the variance (R^2^ = 0.35), suggesting that additional factors likely influence perceptions. According to Rogers’s Diffusion of Innovations theory, attributes such as compatibility, trialability, and social influence often shape adoption decisions in educational contexts [20]. For example, Nezamdoust et al. (2022) found that perceived compatibility and relative advantage significantly influenced nurses’ willingness to adopt mobile health technologies [21]. Faculty attitudes may also be shaped by institutional culture, peer modeling, and leadership endorsement—factors not measured in this study but worth exploring in future research.

Nursing students in our study consistently rated satisfaction and perceived effectiveness of Holopatient simulations higher than faculty, echoing broader findings that immersive technologies boost engagement, confidence, and knowledge retention. For example, a 2024 quasi-experimental study in anatomy education reported 96% satisfaction and significant gains in learning outcomes with immersive VR tools [22]. A narrative review of healthcare education simulations also noted that students “generally expressed positive attitudes toward VR, citing higher satisfaction, engagement, and motivation compared to traditional learning methods” [23]. These results align with research showing that students value realistic, interactive, and self-paced experiences in simulation modalities [24].

Additionally, nursing students’ strong acceptance may stem from their familiarity with digital environments and preference for experiential, technology-enhanced learning, characteristic of Generation Z learners [25]. Immersive mixed-reality tools like Holopatient simulations offer safer, repeatable, and anxiety-reduced spaces for practicing clinical reasoning, benefits well-documented in student-focused VR research [23]. These insights highlight that students appreciate Holopatient experiences both for their pedagogical value and alignment with contemporary learning styles. The findings of this study have meaningful implications for nursing education and clinical preparedness. As Holopatient technology continues to gain traction, its successful integration hinges not only on student readiness but also on faculty engagement and institutional support [26, 27]. The observed gap between student enthusiasm and faculty hesitancy suggests the need for structured faculty development programs focused on digital literacy and simulation pedagogy [28]. Addressing implementation challenges through targeted training, peer mentoring, and access to technical support can enhance faculty confidence and reduce resistance [29]. Moreover, early exposure to digital tools during academic training may foster long-term acceptance of emerging technologies [29]. Future research should explore longitudinal outcomes of Holopatient-based learning on clinical competence, as well as intervention studies that evaluate the effectiveness of faculty training models in improving adoption and instructional quality [30].

Additionally, our findings underscore the importance of comprehensive instructor training and resource allocation, which align with the INACSL Healthcare Simulation Standards of Best Practice®—particularly the Professional Development, Simulation Design, and Facilitation standards [31–33]. These standards recommend formal training in simulation pedagogy, structured scenario planning, and appropriate resource support to ensure high-quality outcomes.

This study presents several strengths, including its comparative design, which enabled direct analysis of differing perceptions between students and faculty using the same simulation technology. The inclusion of multiple analytic approaches—descriptive statistics, subgroup comparisons, and regression modeling—provided a robust framework for identifying both patterns and predictors of perception. Additionally, the study contributes to a relatively limited but growing body of evidence on Holopatient technology in nursing education, especially within settings where adoption is still emerging.

However, certain limitations should be acknowledged. The study was conducted at a single institution, which may limit the generalizability of findings to other educational or cultural contexts. The use of self-reported data introduces the possibility of response bias, and the cross-sectional design prevents the assessment of changes in perceptions over time. Although the questionnaire demonstrated strong internal consistency (Cronbach’s α), it was a researcher-developed tool and lacked formal psychometric validation procedures such as exploratory or confirmatory factor analysis. This raises the possibility that students’ high satisfaction ratings may reflect general enthusiasm for digital learning rather than perceptions specific to Holopatient technology. Additionally, there was an unequal distribution between the two groups, with a larger number of student participants compared to faculty members. This imbalance may have influenced the overall findings, as students’ responses could have disproportionately shaped the results. Future studies with more balanced group sizes are recommended to better capture divergent perspectives.

Furthermore, while the questionnaire demonstrated strong internal consistency, it did not include qualitative components that might have enriched an understanding of the underlying reasons behind faculty and student attitudes. Future studies could address these limitations by employing multi-institutional samples, longitudinal designs, and mixed-methods approaches.

## Conclusions

This study adds new insights to the understanding of how Holopatient technology is perceived differently by students and faculty within nursing education. While students demonstrated high levels of satisfaction and perceived value, faculty members reported lower overall enthusiasm, and those with limited teaching experience reported challenges related to implementation.

The identification of prior simulation exposure and digital literacy as key predictors of positive perception further emphasizes the importance of familiarity and confidence in the successful adoption of emerging technologies. Conversely, the inverse relationship between teaching experience and positive attitudes suggests that longstanding pedagogical habits may influence openness to innovation.

For nursing education programs aiming to implement Holopatient technology effectively, the findings underline the necessity of providing faculty with adequate training, support, and time to adapt. Structured professional development and institutional investments in digital infrastructure are essential to bridging the perception gap and maximizing the benefits of immersive simulation. As technology continues to evolve, longitudinal and interventional studies are needed to assess its sustained educational and clinical impact, especially in resource-limited settings.

To strengthen the generalizability and contextual relevance of findings, future research should involve multi-institutional studies across diverse cultural and educational settings, enabling a broader understanding of how immersive digital tools are perceived and implemented globally.

## Electronic supplementary material

Below is the link to the electronic supplementary material.

Supplementary Material 1

## Data Availability

The datasets used and/or analyzed during the current study are available from the corresponding author upon reasonable request.

## Abbreviations

INACSL: International Nursing Association for Clinical Simulation and Learning
IRB: Institutional Review Board
SD: Standard Deviation
VR: Virtual reality

## References

[1] Alrashidi N, Pasay E, Alrashedi MS, Alqarni AS, Gonzales F, Bassuni EM, et al. Effects of simulation in improving the self-confidence of student nurses in clinical practice: a systematic review. BMC Med Educ. 2023;23:1–12. 37904153 10.1186/s12909-023-04793-1 PMC10614341

[2] Liu K, Zhang W, Li W, Wang T, Zheng Y. Effectiveness of virtual reality in nursing education: a systematic review and meta-analysis. BMC Med Educ. 2023;23:1–10. 37770884 10.1186/s12909-023-04662-x PMC10540340

[3] Rummel L, Qi ZT, Jauny R, Redpath A, Watson S, Solomon B, et al. The effectiveness of augmented reality technology versus traditional teaching methods for undergraduate nursing education. Int J Multiling Biomechatron And Biomed Robot. 2023;4:94–105.

[4] Kang Y, Lee I. The effect of mixed reality-based HoloPatient in problem-based learning contexts. Clin Simul Nurs. 2023;82:101438.

[5] World Health Organization. Simulation in nursing and midwifery education. 2018.

[6] Using cutting-edge technology to revolutionise nursing education | King’s college London. https://www.kcl.ac.uk/news/using-cutting-edge-technology-revolutionise-nursing-education. Accessed 10 Apr 2025

[7] Hologram technology enhances nursing studies at UC -. https://www.canberra.edu.au/about-uc/media/newsroom/2017/september/hologram-technology-enhances-nursing-studies-at-uc. Accessed 10 Apr 2025 University of Canberra.

[8] Minty I, Lawson J, Guha P, Luo X, Malik R, Cerneviciute R, et al. The use of mixed reality technology for the objective assessment of clinical skills: a validation study. BMC Med Educ. 2022;22:1–8. 35999532 10.1186/s12909-022-03701-3 PMC9395785

[9] Saragih ID, Suarilah I, Hsiao CT, Fann WC, Lee BO. Interdisciplinary simulation-based teaching and learning for healthcare professionals: a systematic review and meta-analysis of randomized controlled trials. Nurse Educ Pract. 2024;76. 10.1016/j.nepr.2024.103920 38382335

[10] Son Y, Kang HS, De Gagne JC. Nursing students’ experience of using HoloPatient during the coronavirus disease 2019 pandemic: a qualitative descriptive study. Clin Simul Nurs. 2023;80:9–16. 37101654 10.1016/j.ecns.2023.03.007 PMC10073590

[11] Dicheva NK, Rehman IU, Anwar A, Nasralla MM, Husamaldin L, Aleshaiker S. Digital transformation in nursing Education: a systematic review on computer-aided nursing Education pedagogies, recent advancements and outlook on the Post-COVID-19 Era. IEEE Access. 2023;11:135659–95.

[12] Rincon E, Rodriguez-Guidonet I, Andrade-Pino P, Monfort-Vinuesa C. Mixed reality in undergraduate mental health education: a systematic review. Electron (switz). 2023;12:1019.

[13] Choi MJ, Kim KJ. Effects of team-based mixed reality simulation program in emergency situations. PLoS One. 2024;19. 10.1371/journal.pone.0299832 PMC10903827 38422080

[14] Pregowska A, Osial M, Dolega-Dolegowski D, Kolecki R, Proniewska K. Information and communication technologies combined with mixed reality as supporting tools in medical education. Electronics. 2022;11:3778.

[15] Ahmed R. Exploring barriers to technology integration in nursing education: an unveiling through a qualitative study. 2025. 10.21203/RS.3.RS-6443341/V1.

[16] Tabatabaee SS, Jambarsang S, Keshmiri F. Cognitive load theory in workplace-based learning from the viewpoint of nursing students: application of a path analysis. BMC Med Educ. 2024;24:1–8. 38890747 10.1186/s12909-024-05664-z PMC11186199

[17] Hessler KL, Henderson AM. Interactive learning research: application of cognitive load theory to nursing education. Int J Nurs Educ scholarsh. 2013;10:133–41. 10.1515/ijnes-2012-0029 23813334

[18] Zarakovitis D, Tsoromokos D, Tsaloukidis N, Lazakidou A. Adoption of metaverse technologies in medical and nursing education. Int J Electron healthc. 2024;14:81–90.

[19] Sugar W, Crawley F, Fine B. (PDF) examining teachers’ decisions to adopt new technology. Educational Technology & Society; 2004.

[20] Sahin I. Detailed review of Rogers’ diffusion of innovations theory and educational technology-related studies based on Rogers’ theory. Turk Online J Educ Technol - TOJet. 2006;5:14–23.

[21] Nezamdoust S, Abdekhoda M, Rahmani A. Determinant factors in adopting mobile health application in healthcare by nurses. BMC Med Inf Decis Mak. 2022;22. 10.1186/s12911-022-01784-y PMC8862523 35193552

[22] Jallad ST, Natsheh I, Helo LA, Ibdah DM, Salah A, Muhsen R, et al. Nursing student’s perceptions, satisfaction, and knowledge toward utilizing immersive virtual reality application in human anatomy course: quasi-experimental. Bmc Nurs. 2024;23:1–11. 39198772 10.1186/s12912-024-02254-8 PMC11361164

[23] Houser C, Goodman A, Mack J, Simon B. Efficacy and students’ perceptions of virtual reality in clinical education: a narrative review. Cureus. 2025;17. 10.7759/cureus.82852 PMC12102637 40416184

[24] Kim MJ, Kang HS, De Gagne JC. Nursing students’ perceptions and experiences of using virtual simulation during the COVID-19 Pandemic. Clin Simul Nurs. 2021;60:11. 34249183 10.1016/j.ecns.2021.06.010 PMC8257426

[25] Liu Y, Sun X, Zhang P, Han P, Shao H, Duan X, et al. Generation Z nursing students’ online learning experiences during COVID-19 epidemic: a qualitative study. Heliyon. 2023;9:e14755. 36968654 10.1016/j.heliyon.2023.e14755 PMC10032057

[26] Gause G, Mokgaola IO, Rakhudu MA. Technology usage for teaching and learning in nursing education: an integrative review. Curationis. 2022;45:2261. 35792609 10.4102/curationis.v45i1.2261 PMC9257720

[27] Shon S, Shin H, Rim D, Jeon H. Nursing faculty development program for digital teaching competence. BMC Med Educ. 2024;24:1–10. 38720333 10.1186/s12909-024-05453-8 PMC11080220

[28] Dubois N, Tonus C, Klenkenberg S, Donneau AF, Buléon C, Ghuysen A. Massive open online course: a new strategy for faculty development needs in healthcare simulation. Adv simulation. 2024;9:1–8. 10.1186/s41077-024-00318-y PMC11577793 39568094

[29] Jeffries PR, Dreifuerst KT, Kardong-Edgren S, Hayden J. Faculty development when initiating simulation programs: lessons learned from the national simulation study. J Nurs Regul. 2015;5:17–23.

[30] Taylor I, Bing-Jonsson PC, Finnbakk E, Wangensteen S, Sandvik L, Fagerström L. Development of clinical competence - a longitudinal survey of nurse practitioner students. Bmc Nurs. 2021;20:1–15. 34271923 10.1186/s12912-021-00627-x PMC8283382

[31] Healthcare simulation standards of best Practice®. https://www.inacsl.org/healthcare-simulation-standards. Accessed 6 Jul 2025

[32] Almagharbeh WT, Alfanash HA, Alnawafleh KA, Alasmari AA, Alsaraireh FA, Dreidi MM, Nashwan AJ. Application of artificial intelligence in nursing practice: A qualitative study of Jordanian nurses’ perspectives. Bmc Nurs. 2025;24:42. 10.1186/s12912-024-02658-6. 39863852 10.1186/s12912-024-02658-6 PMC11762109

[33] Almagharbeh WT. The impact of AI-based decision support systems on nursing workflows in critical care units. Int Nurs Rev. 2025;72(1):e13011. 10.1111/inr.13011. 38973347 10.1111/inr.13011

