# Supplementary material for: Holopatient technology in nursing education: a cross-sectional analysis of student and faculty perceptions
Source: BMC Nurs. 2025 Sep 26;24:1192. doi: 10.1186/s12912-025-03856-6 (PMC12465255; doi:10.1186/s12912-025-03856-6)
Supplement: Supplementary file 1 — Supplementary Material 1 [file 12912_2025_3856_MOESM1_ESM.docx]

Questionnaire

| **Domain** | **Item Number** | **Question** |
| --- | --- | --- |
| Perceived Effectiveness | 1 | Holopatient technology enhanced my clinical reasoning skills. |
|  | 2 | Holopatient simulation improved my knowledge retention. |
|  | 3 | I feel better prepared for clinical practice after using holopatient. |
|  | 4 | Holopatient sessions helped me apply theoretical concepts effectively. |
|  | 5 | Holopatient technology improved my critical thinking skills. |
| Overall Satisfaction | 6 | I am satisfied with my experience using holopatient technology. |
|  | 7 | I enjoyed participating in holopatient simulations. |
|  | 8 | I would recommend holopatient simulations to other students/faculty. |
|  | 9 | Holopatient sessions were engaging and motivating. |
|  | 10 | Overall, holopatient technology met my expectations. |
| Ease of Use | 11 | The holopatient technology was easy to navigate. |
|  | 12 | I found the interface of the holopatient system intuitive. |
|  | 13 | Instructions for using the holopatient system were clear and accessible. |
|  | 14 | I was able to use the holopatient technology with minimal technical support. |
|  | 15 | The holopatient sessions were free of major technical issues. |
| Implementation Challenges | 16 | Integrating holopatient technology into the curriculum is challenging. |
|  | 17 | Faculty require additional training to effectively use holopatient tools. |
|  | 18 | Technical limitations make holopatient implementation difficult. |
|  | 19 | Cost considerations hinder the adoption of holopatient technology. |
|  | 20 | Infrastructure and resource limitations affect holopatient use. |
